# Macular vessel density versus ganglion cell complex thickness for detection of early primary open-angle glaucoma

**DOI:** 10.1186/s12886-020-1304-x

**Published:** 2020-01-08

**Authors:** Yiwei Wang, Chen Xin, Meng Li, David L. Swain, Kai Cao, Huaizhou Wang, Ningli Wang

**Affiliations:** 1 Beijing Institute of Ophthalmology, Beijing Tongren Eye Centre, Beijing Tongren Hospital, Capital Medical University, 1 Dongjiaominxiang Street, Dongcheng District, Beijing, 100730 China; 2 Beijing Ophthalmology & Visual Sciences Key Laboratory, 1 Dongjiaominxiang Street, Dongcheng District, Beijing, 100730 China; 3 Department of Ophthalmology, Boston University School of Medicine, Boston, MA 02118 USA; 4 Department of Anatomy and Neurobiology, Boston University School of Medicine, Boston, MA 02118 USA; 5 Department of Ophthalmology, Beijing Tongren Eye Centre, Beijing Tongren Hospital, Capital Medical University, Beijing, 100730 China

**Keywords:** Primary open-angle glaucoma, Pre-perimetric glaucoma, Macular vessel density, Ganglion cell complex, Optical coherence tomography angiography

## Abstract

**Background:**

To evaluate the macular vessel density (VD) and ganglion cell complex (GCC) thickness in pre-perimetric (PPG) and early perimetric primary open-angle glaucoma (PG) eyes, and to compare the diagnostic ability of the two measurements to discriminate PPG and early PG eyes from healthy eyes.

**Methods:**

Seventy-nine eyes in 72 subjects (31 normal, 26 PPG, and 22 early PG eyes) were included in the consecutive case series. Macular VD and GCC thickness were acquired simultaneously using the 6 × 6 mm^2^ high-density AngioRetina scanning mode. Diagnostic abilities were assessed using the area under the receiver operating characteristic curve (AUROC).

**Results:**

Compared to healthy eyes, whole image VD (wiVD) and GCC thickness were significantly lower in PPG and early PG eyes (all *P* < 0.025). The percent reduction of wiVD was lower than that of GCC thickness in early PG eyes (*P* < 0.05), while they were similar in PPG eyes (*P* > 0.05). Regionally, greater VD attenuation and GCC thinning were identified in the perifovea than in the parafovea in both groups (all *P* < 0.05). Moreover, the percent reduction of VD was less than that of GCC thickness in the perifoveal region in PPG eyes (*P* < 0.05). The AUROCs for wiVD and GCC thickness were 0.824 and 0.881, respectively, in PPG eyes (*P* > 0.05), and 0.918 and 0.977, respectively, in early PG eyes (*P* > 0.05).

**Conclusions:**

Macular VD and GCC thickness significantly decreased in PPG and early PG eyes. The perifoveal region appeared to be more vulnerable to macular VD attenuation and GCC thinning in early glaucoma. Our results showed that macular VD measurements may be helpful for detecting and understanding early glaucomatous damage.

## Background

Primary open-angle glaucoma (POAG) is a leading cause of irreversible blindness worldwide [1], and it is characterized by progressive degeneration of retinal ganglion cells (RGCs) and their axons [2]. Many factors are involved in the development of POAG, including vascular insufficiency [3–6]. Since RGCs are nourished by the superficial retinal capillary plexus, it is presumed that decreased superficial retinal microcirculation in the macular region may occur in the course of glaucoma.

Optical coherence tomography angiography (OCTA) is a noninvasive imaging technique that provides reproducible measurements of vasculature in defined segments by separating static signals from motion signals [7, 8]. Recent studies have shown decreased superficial retinal microcirculation in the macular region in eyes with POAG [9–15]. More interestingly, Yarmohammadi et al. [16] reported that macular vessel density (VD) attenuated in the perimetrically intact hemiretina of glaucoma eyes with a single-hemifield defect, suggesting that significant microvasculature alterations in the macular region may precede detectable visual field (VF) defects.

Early glaucomatous damage has been shown to involve the macula [17, 18]. Studies have reported that 25 to 50% of RGCs were lost prior to detectable VF impairment [19, 20]. Therefore, macular measurements may be potential indicators for early detection of glaucoma. Structural parameters in the macular region such as the ganglion cell complex (GCC) or ganglion cell–inner plexiform layer have been found to be thinner in eyes with pre-perimetric glaucoma (PPG) than in healthy eyes [21–25]. However, only a few studies have focused on macular microcirculation changes in early glaucoma [26–30]. Among these studies, fewer have focused on PPG [28–30]. The current study aimed to evaluate macular VD and GCC thickness in eyes with PPG and early perimetric POAG (PG), and to compare the diagnostic abilities of the two parameters in discriminating PPG or early PG eyes from healthy eyes.

## Methods

This observational study was conducted in Beijing Tongren Hospital between January 2018 and May 2018. The study followed the tenets of the Declaration of Helsinki and was approved by the Institutional Review Board of Beijing Tongren Hospital. Informed consent was obtained from all subjects.

Overall inclusion criteria were an age of ≥18 years, open angles under gonioscopy, a best-corrected visual acuity of 0.5 or better, and a spherical equivalent within + 3 to − 6 diopters. The exclusion criteria were a prior intraocular surgery except for uncomplicated cataract or glaucoma surgery; non-glaucomatous optic neuropathy and retinopathy, uveitis, or ocular trauma; diagnosis of Parkinson’s disease, Alzheimer’s disease, or history of stroke; any other known disease that may cause optic neuropathy, retinopathy or VF loss; an unreliable VF; and poor-quality OCTA scans as described later.

Healthy controls were defined as individuals who had an intraocular pressure (IOP) of < 21 mmHg without history of elevated IOP, normal-appearing optic nerve head, intact neuroretinal rim and retinal nerve fiber layer (RNFL), and reliable normal VF. A normal VF was defined as a pattern standard deviation (PSD) within the 95% confidence limit, and a glaucoma hemifield test result within the normal limits by reliable VF test [31]. A reliable VF test was defined as a VF with a false-positive error of < 15%, a false-negative error of < 15%, and a fixation loss of < 33%. PPG in this study was defined based on the presence of a normal anterior segment on a slit-lamp examination, a gonioscopically open angle and glaucomatous optic neuropathy (i.e., optic nerve rim defect (notching or localized thinning), optic disc hemorrhage, and RNFL defect) in the absence of glaucomatous VF abnormalities [7, 30]. Glaucomatous VF abnormalities were defined as follows: (1) PSD outside normal limits (*P* < 0.05) and glaucoma hemifield test outside normal limits; (2) the presence of at least 3 adjacent points within the same hemifield on the pattern deviation probability plot at *P* < 0.05 with at least 1 point at *P* < 0.01. Early PG eyes showed glaucomatous optic neuropathy, open angles under gonioscopy, and typical glaucomatous VF defects corresponding to glaucomatous optic neuropathy. Early glaucoma was defined as 24–2 MD > − 6 dB.

All participants underwent comprehensive ophthalmic examinations, including assessment of best-corrected visual acuity, slit-lamp biomicroscopy, IOP assessment by Goldmann applanation tonometry, gonioscopy, fundus examination, stereoscopic visualization and photography (Kowa, Japan) of optic nerve head, central corneal thickness (CCT) assessment with the Casia SS-1000 OCT (Tomey, Japan), axial length assessment with the Lenstar Optical Biometer (Haag-Streit, USA), perimetry using the Zeiss Humphrey Field Analyzer (Carl Zeiss Meditec Inc., Dublin, California, USA) with the Swedish interactive threshold algorithm standard 24–2 program, and peripapillary RNFL (pRNFL) thickness assessment with the RTVue XR Avanti with AngioVue system (Optovue Inc., Fremont, CA, USA). Systemic blood pressure (BP) was measured with an Omron automatic BP instrument in an upright sitting position. Mean arterial pressure (MAP) was calculated as follow: MAP = diastolic BP + 1/3 (systolic BP - diastolic BP). Mean ocular perfusion pressure (MOPP) was calculated based on the following equation: MOPP = 2 x (MAP - IOP) / 3.

The macular VD and GCC thickness were acquired simultaneously using the RTVue XR Avanti with AngioVue system by the same operator (W.Y.W). The software version was 2017.1.0.155. The 6 × 6 mm^2^ high-density AngioRetina scanning mode with the ETDRS segmentation algorithm was used in this study. VD was defined as the percentage of the area occupied by vessels in the defined area [32]. We focused on the “superficial vascular complex” (a slab defined by the Angiovue software extending from the internal limiting membrane to the inner plexiform layer - 10 mm) in the macular region [15]. The whole image vessel density (wiVD) was measured in the entire 6 × 6 mm^2^ image. In addition, the retinal map was segmented using three concentric rings with diameters of 1, 3, and 6 mm. The intermediate (the parafoveal region) and outer (the perifoveal region) rings were divided into 4 sectors of 90 degrees each (para−/peri-nasal, para−/peri-inferior, para−/peri-superior, and para−/peri-temporal sectors). The superior-hemifield and inferior-hemifield VD were obtained by dividing the scan area across the horizontal meridian. GCC thickness was acquired using the 6 × 6 mm^2^ high-density AngioRetina scanning mode and measured from the inner limiting membrane (ILM) to the posterior boundary of the inner plexiform layer. The corresponding segmented regions listed above were also used for GCC thickness analysis. The percent reduction of macular VD and GCC thickness was calculated using the following equation: (mean value for normal eyes − mean value for glaucomatous eyes) / mean value for normal eyes. The image quality of all OCT angiograms was assessed. Scans with the presence of one of the following issues were excluded from analysis: a signal strength index (SSI) below 50, residual motion artefacts and a weak local signal due to vitreous opacity.

Statistical analyses were performed using SPSS (v20.0; IBM SPSS Inc., Chicago, USA), MedCalc (v15.2.2; MedCalc Software bvba, Ostend, Belgium) and R statistical computing package (v3.6.0; R Foundation for Statistical Computing, Vienna, Austria). Categorical variables were compared using Chi-square test. Age was compared using the Mann-Whitney test. Mixed effects models were used to compare the ocular characteristics. Univariate and multivariate analyses in mixed effects model were used to compare the macular VD and GCC thickness parameters between PPG, early PG, and healthy eyes. Potential confounding factors, such as age, sex, SSI and any ophthalmic characteristics were included in multivariate models if the *P* value was < 0.05 in univariate analysis. The clustered Wilcoxon signed rank test using the Rosner-Glynn-Lee method was used to compare the percent reduction of the macular VD and GCC thickness within each individual group. The Spearman correlation and mixed effects models were used to assess the associations between macular VD, GCC thickness and other variables (pRNFL, MD and PSD). The diagnostic ability was evaluated by the area under the receiver operating characteristic curve (AUROC). The DeLong method was used for comparisons between AUROCs. Statistical significance was set at two-tailed *P* < 0.05. Bonferroni-corrected significance level was used for multiple comparisons.

## Results

A total of 79 eyes (31 normal, 26 PPG and 22 early PG eyes) from 72 subjects were included for analysis in this study. The demographics and ocular characteristics are summarized in Additional file 1. The age, sex of the subjects, prevalence of a history of hypertension, usage rate of hypotensive drugs, prevalence of diabetes history, usage rate of diabetes drugs, MOPP, CCT, and axial length were not significantly different between the normal and PPG groups, or between the normal and early PG groups (all *P* > 0.025), except that the normal and PPG groups differed by sex (*P* = 0.019). The difference in the usage rate of hypotensive eye drops between the PPG and early PG groups was not statistically significant (*P* > 0.025). The average, superior-hemifield, and inferior-hemifield pRNFL in PPG and early PG eyes were significantly thinner than that in normal eyes (all *P* < 0.001).

**Fig. 1:**
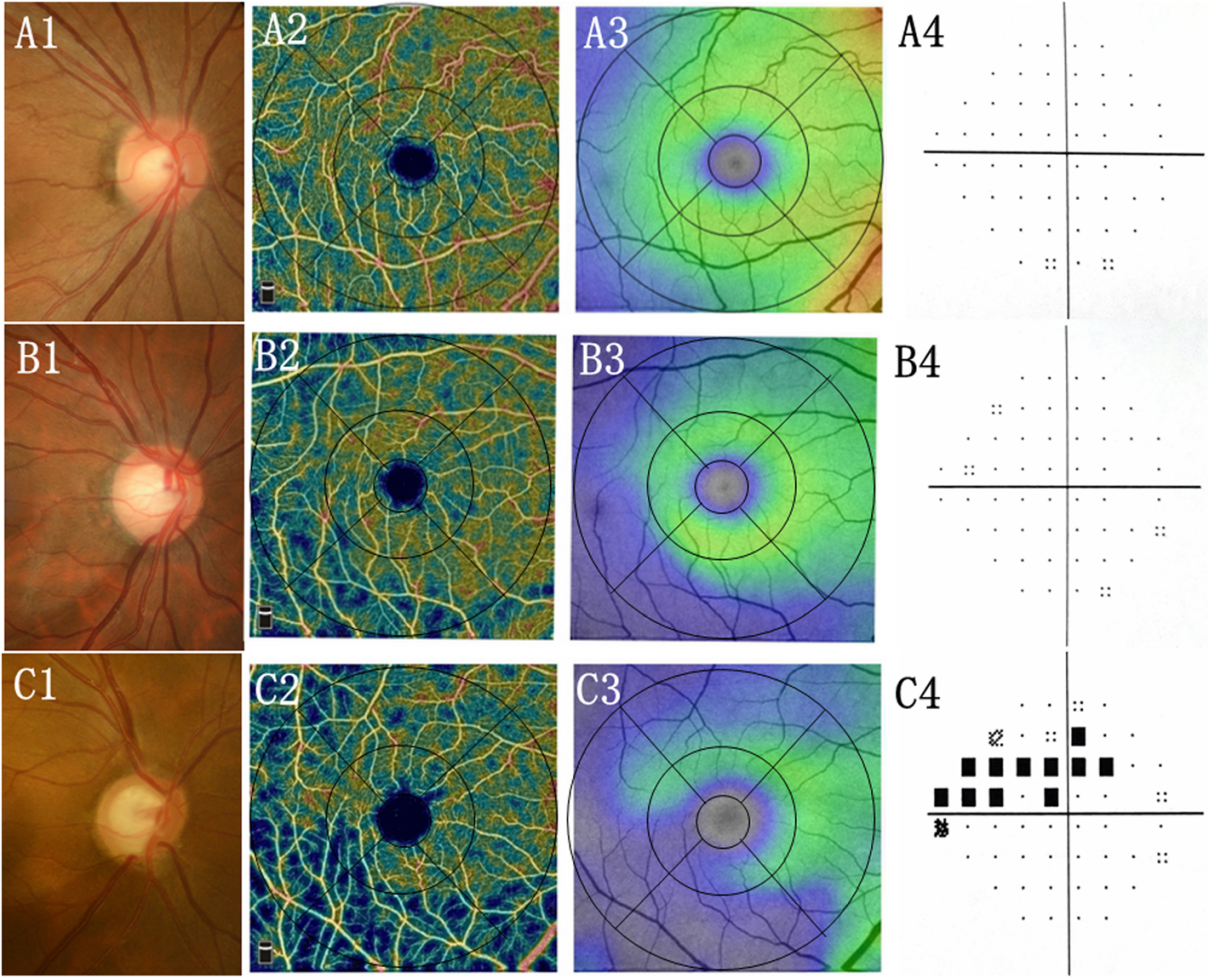
Examples of eyes included in this study. A Normal-appearing optic nerve head with intact rim and RNFL (A1), intact GCC (A3), dense macular microvascular network (A2), and normal VF (A4) in normal eyes. Enlarged vertical cup-to-disc ratio with focal rim notching and RNFL defect (B1, C1), focal defects of macular microvascular network (B2, C2) matching the location of GCC loss (B3, C3) with normal VF (B4) in pre-perimetric glaucoma or with typical glaucomatous VF defects (C4) in early perimetric glaucoma. GCC, ganglion cell complex thickness. RNFL, peripapillary retinal nerve fiber layer; VF, visual field

The macular VD and GCC thickness parameters are presented in Tables 1 and 2, respectively. The SSI in the normal group was significantly higher than that in the PPG (69.3 ± 5.9 vs. 63.9 ± 6.0, *P* = 0.001) and early PG groups (69.3 ± 5.9 vs. 65.1 ± 5.6, *P* = 0.013). Macular wiVD (46.9% ± 3.6% in PPG eyes; 43.1% ± 5.3% in early PG eyes) and GCC thickness (92.4 ± 7.9 in PPG eyes; 80.7 ± 10.9 in early PG eyes) were significantly lower in the PPG and early PG groups than in the control group (50.9% ± 2.8% for wiVD; 103.4 ± 5.6 for GCC thickness) (all *P* < 0.025 in both univariate and multivariate analyses). In terms of regional measurements, the VD and GCC thickness parameters in PPG eyes were less than corresponding parameters in normal eyes in the multivariate analysis model with adjustment for age, gender, and SSI (all *P* < 0.025), except for VD in the parafoveal region and in peri-temporal and peri-nasal sectors. In contrast, all the VD and GCC thickness parameters in the early PG group were significantly smaller compared to normal group in the multivariate analysis model (all *P* < 0.025). Examples of macular VD maps and GCC maps from a healthy eye (Fig. 1a), an eye with PPG (Fig. 1b), and an eye with early PG (Fig. 1c) are shown.

**Table 1:** Macular VD in normal, pre-perimetric and early perimetric glaucoma eyes (Mean ± SD)

	Macular VD (%)			*P* value (univariate, multivariate)	
	normal	PPG	early PG	a	b
wi	50.9 ± 2.8	46.9 ± 3.6	43.1 ± 5.3	< 0.001, 0.002	< 0.001, < 0.001
wi SH	51.0 ± 2.9	47.5 ± 3.7	44.2 ± 5.8	< 0.001, 0.026	< 0.001, < 0.001
wi IH	50.9 ± 2.8	46.4 ± 3.9	41.9 ± 5.7	< 0.001, < 0.001	< 0.001, < 0.001
Regional					
Para	52.4 ± 3.3	49.7 ± 5.2	46.5 ± 7.2	0.022, 0.333	< 0.001, 0.004
Para T	52.4 ± 3.3	50.6 ± 4.0	46.0 ± 7.9	0.066, 0.846	< 0.001, 0.004
Para S	53.2 ± 4.3	50.5 ± 6.0	48.3 ± 6.4	0.046, 0.280	0.001, 0.019
Para N	52.0 ± 3.1	47.7 ± 6.8	45.9 ± 7.5	0.003, 0.052	< 0.001, 0.002
Para I	51.9 ± 4.0	50.1 ± 5.6	45.9 ± 8.1	0.171, 0.851	0.001, 0.013
Peri	51.9 ± 2.8	47.8 ± 3.6	43.6 ± 5.6	< 0.001, 0.002	< 0.001, < 0.001
Peri T	46.9 ± 3.4	43.6 ± 3.7	40.2 ± 4.5	0.001, 0.116	< 0.001, < 0.001
Peri S	52.5 ± 3.2	48.1 ± 3.9	44.5 ± 6.6	< 0.001, 0.009	< 0.001, < 0.001
Peri N	55.9 ± 2.7	53.1 ± 3.5	48.3 ± 7.2	0.001, 0.026	< 0.001, < 0.001
Peri I	52.3 ± 3.0	46.5 ± 4.6	41.1 ± 5.9	< 0.001, < 0.001	< 0.001, < 0.001

Univariate and multivariate analyses using a mixed effects model that was adjusted for age, sex, and SSI were used for comparison. The Bonferroni-corrected significance level (0.025) was used for multiple comparisons

*VD* Vessel density, *PPG* Pre-perimetric glaucoma, *PG* Perimetric glaucoma, *wi* Whole image, *SH* superior hemifield, *IH* Inferior hemifield, *Para* Parafoveal, *Peri* Perifoveal, *T* Temporal, *S* Superior, *N* Nasal, *I* Inferior

a. Comparison between the PPG and normal group

b. Comparison between the early PG and normal group

**Table 2:** GCC thickness in normal, pre-perimetric and early perimetric glaucoma eyes (Mean ± SD)

	GCC thickness (μm)			*P value (*univariate, multivariate*)*	
	normal	PPG	early PG	a	b
Global	103.4 ± 5.6	92.4 ± 7.9	80.7 ± 10.9	< 0.001, < 0.001	< 0.001, < 0.001
Global SH	103.2 ± 5.6	93.6 ± 8.2	84.2 ± 12.3	< 0.001, < 0.001	< 0.001, 0.001
Global IH	103.6 ± 6.0	91.2 ± 9.4	77.1 ± 12.4	< 0.001, < 0.001	< 0.001, < 0.001
Regional					
Para	110.4 ± 8.5	103.9 ± 9.7	89.2 ± 15.9	0.010, < 0.001	< 0.001, < 0.001
Para T	102.5 ± 7.9	96.3 ± 10.5	81.3 ± 13.5	0.015, 0.001	< 0.001, < 0.001
Para S	113.2 ± 9.1	105.6 ± 10.7	93.4 ± 16.0	0.005, 0.001	< 0.001, 0.001
Para N	111.6 ± 9.5	107.1 ± 8.0	92.5 ± 18.7	0.058, < 0.001	< 0.001, < 0.001
Para I	114.3 ± 8.6	106.8 ± 11.5	89.5 ± 20.3	0.007, < 0.001	< 0.001, < 0.001
Peri	103.2 ± 5.7	90.6 ± 8.0	79.5 ± 10.5	< 0.001, < 0.001	< 0.001, < 0.001
Peri T	87.2 ± 4.2	76.4 ± 7.7	68.2 ± 7.9	< 0.001, < 0.001	< 0.001, < 0.001
Peri S	103.1 ± 7.2	90.0 ± 9.8	80.9 ± 14.1	< 0.001, < 0.001	< 0.001, 0.002
Peri N	121.1 ± 7.7	111.2 ± 8.8	98.9 ± 14.4	< 0.001, < 0.001	< 0.001, 0.001
Peri I	101.6 ± 7.3	84.9 ± 11.7	70.1 ± 13.0	< 0.001, < 0.001	< 0.001, < 0.001

Univariate and multivariate analyses using mixed effects model with adjustment for age, gender, and SSI were used to for comparison. The Bonferroni-corrected significance level (0.025) was used for multiple comparisons

*GCC* Ganglion cell complex, *PPG* Pre-perimetric glaucoma, *PG* Perimetric glaucoma, *SH* Superior hemifield, *IH* Inferior hemifield, *para* Parafoveal, *peri* Perifoveal, *T* Temporal, *S* Superior, *N* Nasal, *I* Inferior

a. Comparison between the PPG and normal group

b. Comparison between the early PG and normal group

The percent reduction of macular VD and GCC thickness in the PPG and early PG groups are displayed in Table 3. The percent reduction of wiVD was less than that of GCC thickness in early PG eyes (15.4% vs. 22.0%, *P* = 0.001), while the percent reductions were comparable in PPG eyes (7.8% vs. 10.6%, *P* = 0.117). In the PPG group, the percent reduction of VD was significantly less than that of GCC thickness in the perifoveal region (7.9% vs. 12.2%, *P* = 0.019), while they were similar in the parafoveal region (5.1% vs. 5.9%, *P* = 0.338). In the early PG group, the percent reduction of VD was significantly less than that of GCC thickness in both the perifoveal (16.1% vs. 22.9%, *P* = 0.001) and parafoveal regions (7.9% vs. 12.2%, *P* < 0.05). Moreover, VD attenuation and GCC thinning were more severe in the perifoveal region compared to the parafoveal region in both groups (all *P* < 0.05). The percent reduction of VD and GCC thickness in the early PG group were greater than that in the PPG group (all *P* < 0.05), except in the para-superior (*P* = 0.126) and para-nasal sectors (*P* = 0.308).

**Table 3:** Percent reduction of macular VD and GCC thickness in pre-perimetric and early perimetric glaucoma eyes (mean, 95% confidence interval)

	Pre-perimetric glaucoma			Early perimetric glaucoma		
	VD (%)	GCC Thickness (%)	*P*	VD (%)	GCC Thickness (%)	*P*
Global *^,^ **	7.8 (5.0, 10.7)	10.6 (7.5, 13.7)	0.117	15.4 (10.6, 20.0)	22.0 (17.3, 26.6)	0.001
Global SH *^,^ **	6.9 (4.0, 9.8)	9.3 (6.1, 12.5)	0.096	13.3 (8.2, 18.4)	18.4 (13.1, 23.6)	0.004
Global IH *^,^ **	8.9 (5.7, 12.0)	11.7 (8.0, 15.4)	0.152	17.6 (12.7, 22.6)	25.4 (20.1, 30.7)	0.001
Regional						
Para *^,^ **	5.1 (1.1, 9.0)	5.9 (2.3, 9.4)	0.338	11.2 (5.2, 17.3)	19.2 (12.9, 25.6)	0.001
Peri *^,^ **	7.9 (5.1, 10.7)	12.2 (9.1, 15.3)	0.019	16.1 (11.3, 20.8)	22.9 (18.4, 27.4)	0.001
	*P* = 0.020	*P* < 0.001		*P* = 0.003	*P* = 0.040	
Para T *^,^ **	3.5 (0.4, 6.5)	6.0 (1.9, 10.2)	0.128	12.2 (5.6, 18.9)	20.7 (14.9, 26.6)	0.001
Para S **	5.2 (0.6, 9.7)	6.7 (2.9, 10.5)	0.234	9.2 (3.9, 14.6)	17.5 (11.2, 23.7)	0.001
Para N **	8.2 (3.0, 13.4)	4.0 (1.2, 7.0)	0.873	11.8 (5.4, 18.2)	17.1 (9.7, 24.6)	0.018
Para I *^,^ **	3.4 (−0.01, 7.7)	6.6 (2.5, 10.6)	0.206	11.5 (4.6, 18.4)	21.7 (13.8, 29.6)	0.003
Peri T *^,^ **	7.1 (4.0, 10.3)	12.4 (8.8, 16.0)	0.014	14.2 (9.9, 18.5)	21.8 (17.8, 25.8)	0.001
Peri S *^,^ **	8.4 (5.4, 11.5)	12.8 (9.0, 16.7)	0.026	15.1 (9.6, 20.7)	21.5 (15.4, 27.6)	0.005
Peri N *^,^ **	5.0 (2.5, 7.5)	8.1 (5.2, 11.1)	0.103	13.6 (7.8, 19.3)	18.3 (13.0, 23.6)	0.013
Peri I *^,^ **	11.0 (7.5, 14.5)	16.5 (11.8, 21.5)	0.023	21.3 (16.3, 26.3)	31.0 (25.3, 36.7)	0.001

A clustered Wilcoxon signed rank test using the Rosner-Glynn-Lee method was used to compare the percent reduction of macular VD or GCC thickness between different areas within each individual group

*P, P* values between macular VD and GCC thickness or between the different regions in the individual group

*VD* Vessel density, *GCC* Ganglion cell complex, *SH* Superior hemifield, *IH* Inferior hemifield, *para* Parafoveal, *peri* Perifoveal, *T* Temporal, *S* superior, *N* Nasal, *I* Inferior

Multivariate analysis using a mixed effects model that was adjusted for age, sex, and SSI was used for the comparison between the PPG and early PG groups in terms of the percent reduction of VD (*, *P* < 0.05) and GCC thickness (**, *P* < 0.05)

**Fig. 2:**
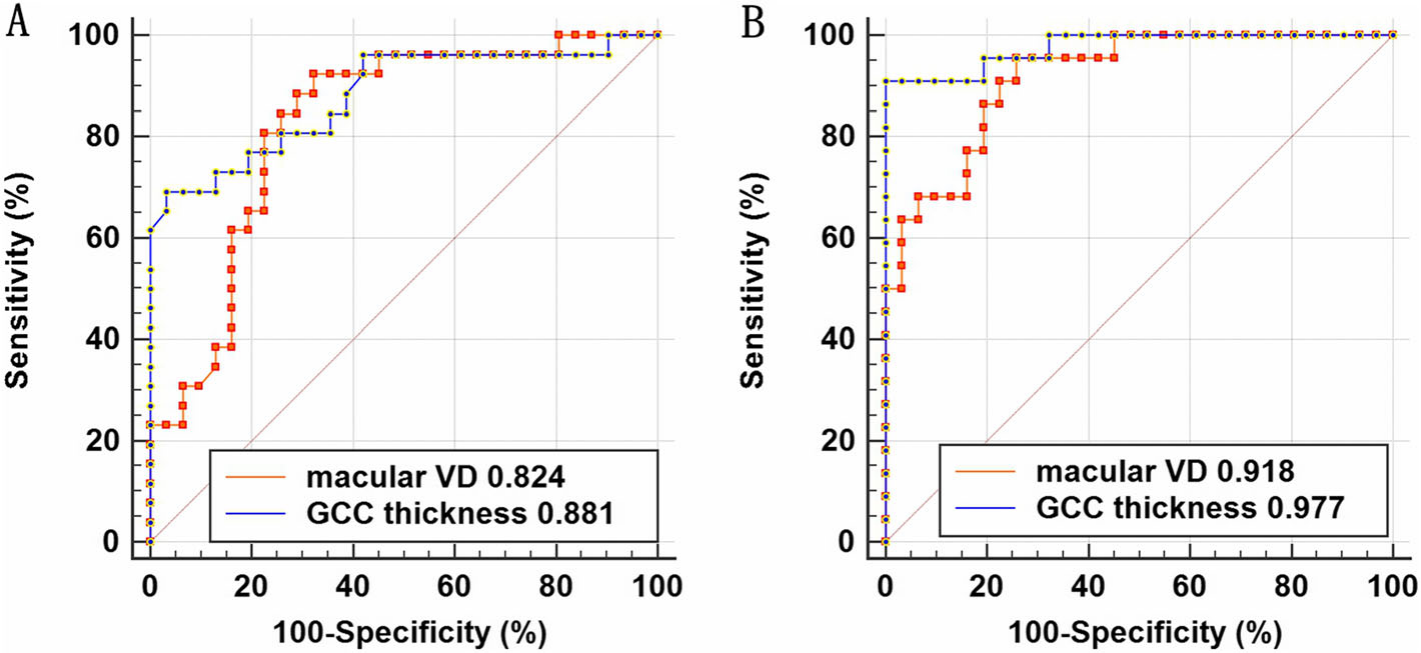
AUROC values for macular VD and GCC thickness. Macular VD showed a lower AUROC value than did GCC thickness in the pre-perimetric glaucoma (A) and early perimetric glaucoma groups (B), but the differences were not statistically significant (both *P* > 0.05). AUROC, area under the receiver operating characteristic curve; VD, vessel density; GCC, ganglion cell complex

The diagnostic ability of macular VD and GCC thickness in differentiating healthy controls from PPG or early PG eyes, as measured by the AUROC, is demonstrated in Table 4 and Fig. 2. AUROC values for the macular VD parameters were comparable to that of the corresponding GCC thickness parameters in both the PPG and early PG groups (all *P* > 0.05). Globally, the AUROC values for wiVD and GCC thickness were 0.824 and 0.881 in the PPG group, respectively, and 0.918 and 0.977 in the early PG group, respectively. As to regional measurements, the perifoveal parameters had greater AUROC values than did the corresponding parafoveal parameters in the PPG group (both *P* < 0.05). In the PG group, the AUROCs of GCC thickness in the perifoveal and parafoveal regions were comparable (*P* = 0.261), while the perifoveal VD showed a significantly better (*P* < 0.05) AUROC than did the parafoveal VD.

**Table 4:** Diagnostic ability of macular vessel density and GCC thickness parameters in differentiating pre-perimetric /early perimetric glaucoma eyes from normal eyes (mean, 95% confidence interval)

	Normal vs. pre-perimetric glaucoma			Normal vs. early perimetric glaucoma		
	VD	GCC Thickness	*P*^a^	VD	GCC Thickness	*P*^a^
wi	0.824 (0.701, 0.912)	0.881 (0.768, 0.952)	0.419	0.918 (0.809, 0.976)	0.977 (0.892, 0.999)	0.109
para	0.635 (0.497, 0.758)	0.694 (0.557, 0.809)	0.580	0.780 (0.645, 0.882)	0.902 (0.788, 0.966)	0.094
peri	0.819 (0.694, 0.908)	0.905 (0.798, 0.967)	0.157	0.918 (0.809, 0.976)	0.959 (0.865, 0.994)	0.286
*P*^b^	< 0.001	< 0.001		0.005	0.261	

*VD* Vessel density, *GCC* Ganglion cell complex, *wi* Whole image, *para* Parafoveal, *peri* Perifoveal

^a^Comparison between the AUROCs for macular VD and GCC thickness in the individual group using Delong’s method

^b^Comparison of the AUROCs for each individual parameter between the parafoveal and perifoveal regions using Delong’s method

The associations among wiVD, GCC thickness, pRNFL thickness, VF MD, and VF PSD are summarized in Additional file 2 and Additional file 3. Spearman correlation analysis showed that wiVD was negatively correlated with VF PSD and positively correlated with GCC thickness, pRNFL thickness, and VF MD in all glaucoma eyes. GCC thickness was negatively correlated with VF PSD and positively correlated with pRNFL thickness and VF MD (Additional file 2). Similar correlations were identified in mixed effects models that were adjusted for age, sex, and SSI (Additional file 3).

## Discussion

In the human retina, RGCs are most densely located at the macula with approximately 50% of the being concentrated within 4.5 mm of the foveal center [33]. Thus, the changes of RGCs in terms of structure or microvasculature parameters in the macula may be sensitive indicators for glaucoma detection. In the current study, we evaluated macular VD and GCC thickness in healthy, PPG and early PG eyes. Our main findings were as follows: (1) A stepwise decrease of wiVD and GCC thickness was identified from normal eyes to PPG eyes to early PG eyes; (2) The diagnostic ability of macular VD is comparable to that of GCC thickness in detecting PPG or early PG; and (3) The percent reductions of VD and GCC thickness in the perifoveal region were greater than that in the parafoveal region in both groups.

Evaluations of the macular VD parameters in PPG eyes were not extensive [8, 26, 29, 30]. Our study showed significant macular microvasculature dropout in PPG and early PG eyes, which was supported by Hou et al. [30] and Poli et al. [29]. In contrast, other studies did not show a significant decrease in macular VD [8, 26]. The discrepancy may be partly attributed to the difference in glaucoma severity among the studies, as GCC atrophy was found in PPG eyes in our study, but remained unchanged in Triolo et al.’s [8] study. Scan sizes may also contribute to an inconsistency in results. Chen et al. [13] reported that no significant difference in the VD was found in the parafoveal area between glaucoma and healthy eyes, while the average VD value in the whole 6 × 6 mm^2^ scanning field was lower in glaucoma eyes. Our regional analysis showed that the perifoveal region seemed more vulnerable to VD attenuation in PPG eyes; thus, the larger scan size used in our study appears more likely to cover the affected area.

Previous studies have reported that the AUROC values for macular VD that ranged from 0.562 to 0.800 for distinguishing between early glaucoma and normal eyes [26, 27, 30, 34]. However, little is known about the diagnostic ability of macular VD in detecting PPG. In our study, wiVD had good diagnostic ability in detecting PPG and early PG eyes (AUROC: 0.824 and 0.918, respectively), which was better than that presented in a previous study [30]. The improved diagnostic ability may be due to the advantage of the larger scan size that we used, as previously mentioned. In addition, macular measurements may offer some advantages, such as less inter-individual structural variability and insusceptibility to the impacts of disc tilting and large peripapillary atrophy [35]. Thus, macular VD measurement may be a supplementary tool for identifying early glaucomatous damage. The AUROCs of wiVD and GCC thickness were comparable in our study, which is consistent with Hou et al.’s results [30]. However, other studies have reported that macular VD performed worse than GCC thickness in differentiating between glaucoma from healthy eyes [9, 13, 15, 36]. The inconsistency may be attributed to the fact that the glaucoma severities differed from our study or the fact that the regions of interest for macular VD measurement and GCC thickness were not the same.

Glaucoma usually starts as focal lesions [2]. The regional mapping of the OCT/OCTA measurements may help us understand early glaucomatous changes. Lin et al. [37] reported that the macular RNFL and macular ganglion cell layer thickness in PPG eyes decreased more in the perifoveal region than in the parafoveal region. However, to the best of our knowledge, there is no study comparing vessel densities in the perifoveal region and parafoveal region in PPG eyes. In this study, we found that VD attenuation and GCC thinning were greater in the perifoveal region in both groups. The peri-inferior sector appeared most vulnerable to glaucomatous damage, which was concordant with the findings of Hood et al. [18, 38]. In addition, we found that the percent reduction of GCC thickness was greater than that of macular VD in eyes with PPG, an earlier course than what reported by Hou et al. [30]. One possible explanation for the mismatch between neural atrophy and vascular insufficiency is that microvascular dropouts can be secondary to neurodegeneration, such as GCC thinning. However, given the sensitivities of the imaging device to detect various parameters may be different, we cannot completely exclude the possibility that a less prominent and undetectable hemodynamic deficiency may occur prior to RGC degeneration. Additional longitudinal studies are required to investigate this issue.

The strengths of the current study included the following: First, macular VD and GCC thickness were acquired over the same scan size, which allowed a fair comparison between the two measurements for detection of glaucoma. Second, we used the AngioRetina HD 6 × 6 mm^2^ mode with the ETDRS segmentation algorithm, which allowed us to evaluate focal changes in the early course of glaucoma. However, there were some limitations to our study. First, the sample size was small. Second, it is impossible to clarify the temporal sequence of macular VD attenuation and GCC thinning due to the cross-sectional design. Third, although the subjects were younger in our study, age has been shown not to affect macular VD measurements [39]. However, we cannot completely rule out the impact of other potential confounding factors, such as systemic disease or hypotensive eye drops, on the VD measurements.

## Conclusions

The current study demonstrated that wiVD and GCC thickness significantly decreased in PPG and early PG eyes compared to controls. The perifoveal region appeared more vulnerable to early glaucomatous damage. The diagnostic accuracy of macular VD was comparable to that of GCC thickness in distinguishing eyes with PPG or early PG from healthy eyes. Overall, macular VD measurements with the larger scan size may show promise to detect early glaucoma, and the regional mapping may be helpful for understanding early glaucomatous damage.

## Supplementary information

**Additional file 1.** The demographic and ocular characteristics of the subjects.

**Additional file 2.** Spearman correlation coefficient matrix on wiVD, GCC thickness, pRNFL thickness, VF MD, and VF PSD.

**Additional file 3.** Mixed-effect model analysis of macular VD and GCC thickness.

## Data Availability

All data included in this study are available from the corresponding author on reasonable request.

## Abbreviations

AL: Axial length
AUROC: Area under the receiver operating characteristic curve
BP: Blood pressure
CCT: Central corneal thickness
IOP: Intraocular pressure
MD: Mean deviation
MOPP: Mean ocular perfusion pressure
OCT: Optical coherence tomography
OCTA: Optical coherence tomography angiography
PG: Periperimetric glaucoma
POAG: Primary open-angle glaucoma
PPG: Pre-perimetric glaucoma
PSD: Pattern standard deviation
RGC: Retinal ganglion cell
RNFL: Retinal nerve fiber layer
SSI: Signal strength index
VD: Vessel density
VF: Visual field
wiVD: Whole image vessel density
