# Supplementary figures and images for: Macular vessel density versus ganglion cell complex thickness for detection of early primary open-angle glaucoma
Source: BMC Ophthalmol. 2020 Jan 8;20:17. doi: 10.1186/s12886-020-1304-x (PMC6950925; doi:10.1186/s12886-020-1304-x)

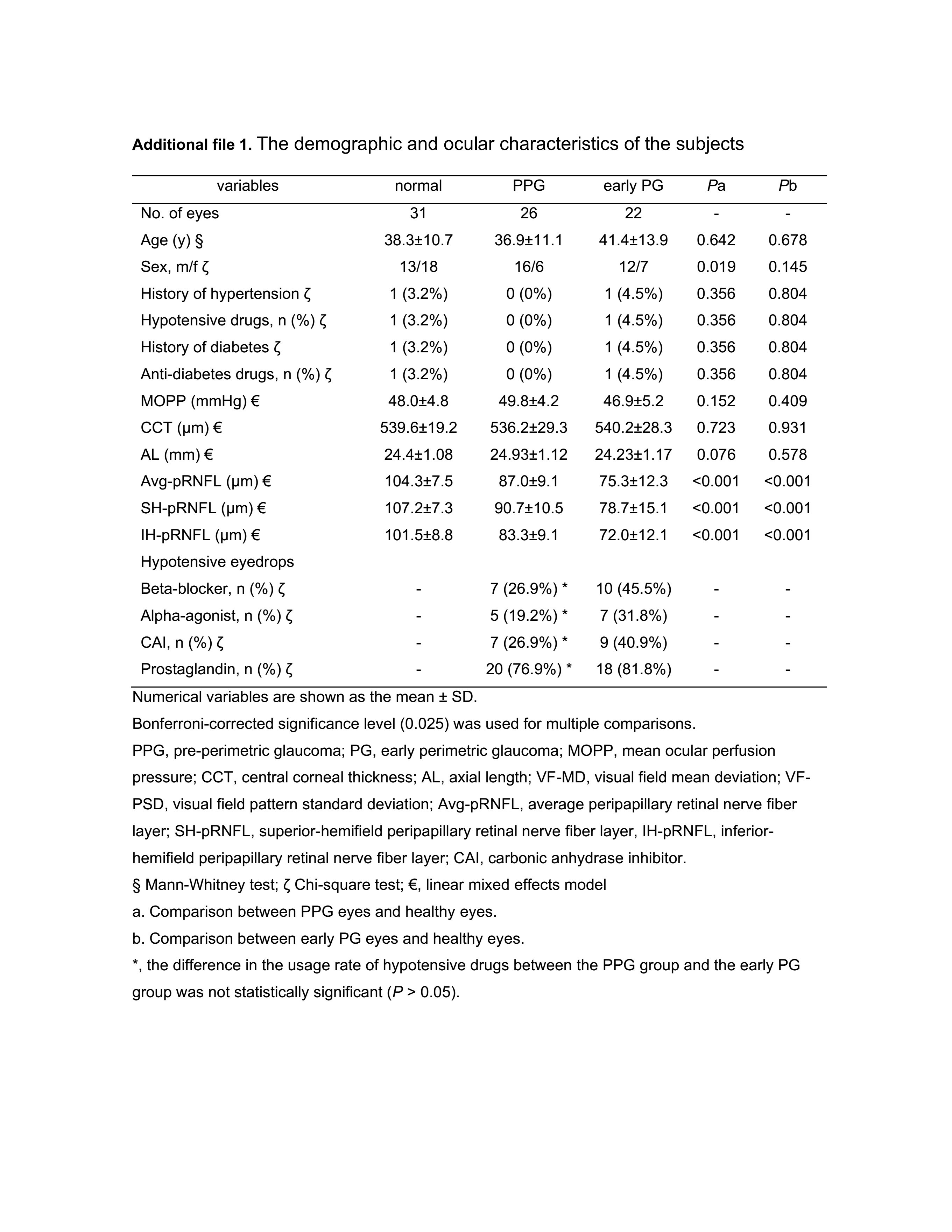

Supplement: Supplementary file 1 — Additional file 1. The demographic and ocular characteristics of the subjects. [file 12886_2020_1304_MOESM1_ESM.tiff]

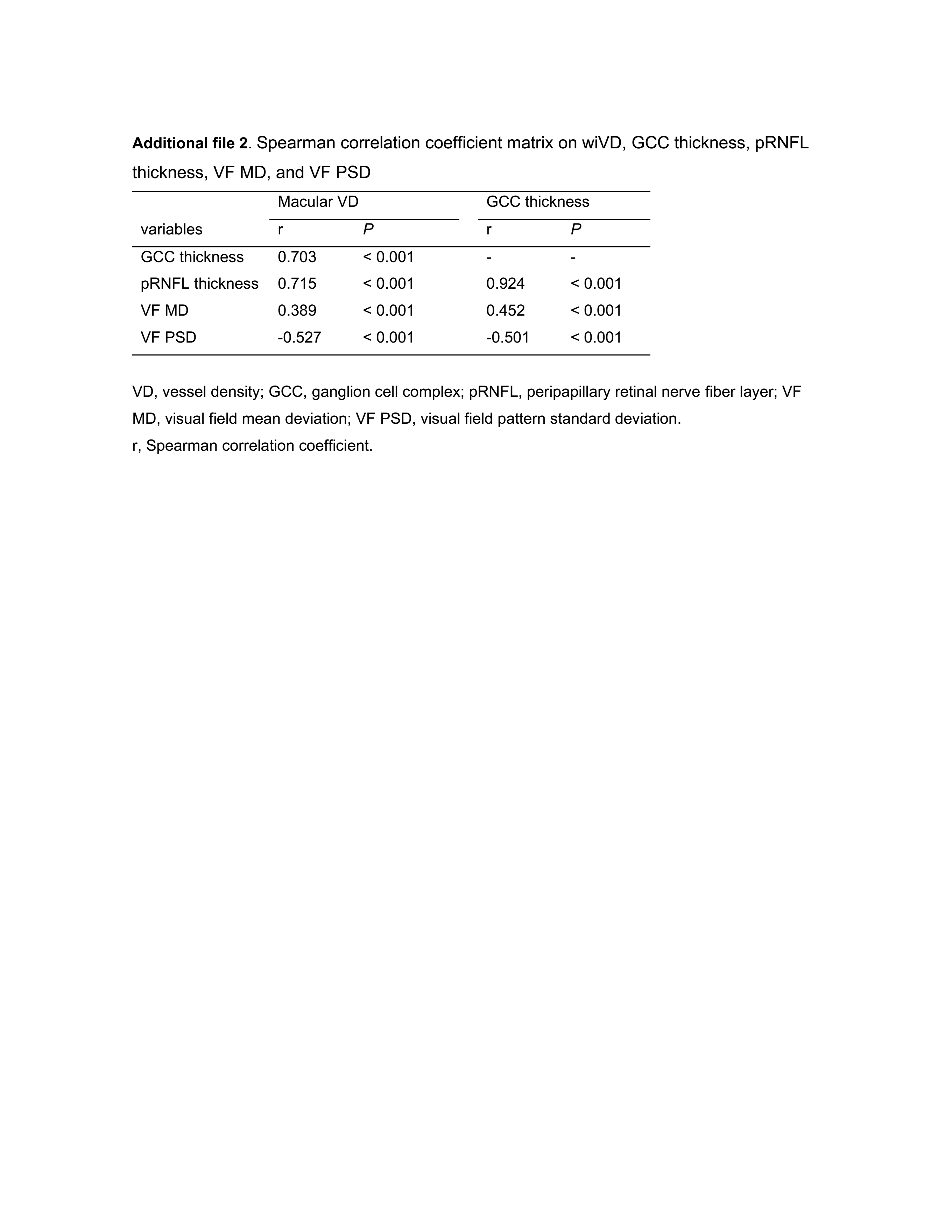

Supplement: Supplementary file 2 — Additional file 2. Spearman correlation coefficient matrix on wiVD, GCC thickness, pRNFL thickness, VF MD, and VF PSD. [file 12886_2020_1304_MOESM2_ESM.tiff]

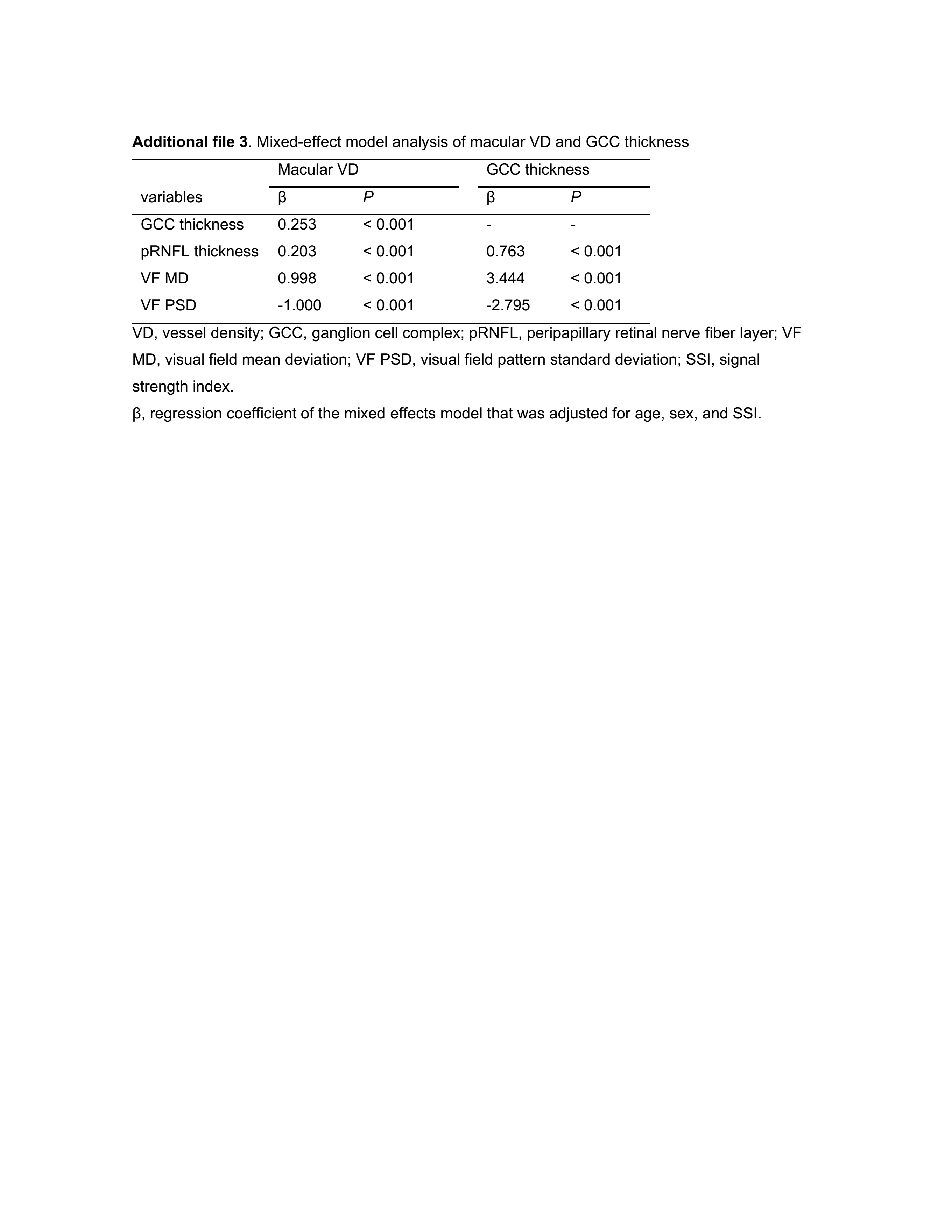

Supplement: Supplementary file 3 — Additional file 3. Mixed-effect model analysis of macular VD and GCC thickness. [file 12886_2020_1304_MOESM3_ESM.tiff]
